# Choosing Wisely recommendations in oncology: a scoping review

**DOI:** 10.1007/s00520-026-10437-z

**Published:** 2026-03-04

**Authors:** Fabiola Vasconcelos Alves, Elaine Barros Ferreira, Fabio Ynoe de Moraes, Safiya Karim, Paula Elaine Diniz dos Reis

**Affiliations:** 1 Faculty of Health Sciences, University of Brasília, Brasília, DF Brazil; 2 Department of Oncology, Queen’s University, Kingston, ON Canada; 3 Department of Oncology, University of Calgary, Calgary, AB Canada

**Keywords:** Choosing wisely, Value-based health care, Oncology, Cancer, Scoping review

## Abstract

Choosing Wisely (CW) campaign addresses the global challenge of reducing waste in healthcare by discouraging unnecessary interventions, minimizing harm, and promoting evidence-based care. Barriers like limited awareness among healthcare professionals and the lack of global mapping of recommendations hinder its implementation. We conducted a comprehensive scoping review mapping the literature on CW recommendations in oncology from 2012 to 2024. A total of 220 recommendations were gathered from 19 studies and 20 recognized international websites. These recommendations explored the importance of personalizing screening, staging, and treatment based on individual risk factors, such as life expectancy and biomarker status. The findings suggest a global shift toward patient-centered oncology care, focusing on personalized, evidence-based treatments and minimizing unnecessary interventions. Developing recommendations based on resource-stratified guidelines is vital for improving the adoption of CW, particularly in low- and middle-income countries, as it ensures that recommendations are aligned with local healthcare needs and available resources.

**Supplementary Information:**

The online version contains supplementary material available at 10.1007/s00520-026-10437-z.

## Introduction

As healthcare systems worldwide strive for sustainability, addressing waste within healthcare services has emerged as a critical challenge [1]. In many countries, unnecessary healthcare interventions remain widespread, contributing to excessive spending, including up to $101 billion annually in the United States of America (USA) alone [2]. The overuse of diagnostic tests and therapeutic procedures not only poses risks to patient safety but also results in inefficient resource allocation [3].

In this regard, the American Board of Internal Medicine (ABIM) launched the Choosing Wisely (CW) campaign in 2012 to reduce inappropriate procedures in clinical practice. This initiative seeks to enhance the quality of healthcare by discouraging unnecessary exams, procedures, and treatments. Since its inception, it has gained significant momentum, with over 80 medical societies in approximately 20 countries adopting its principles [4]. In the field of oncology, the CW campaign originated in the USA and has since been embraced by countries such as Canada, India, various African nations, and Brazil [5–9]. Each campaign identifies specific practices that physicians should question to avoid unnecessary care for cancer patients. While the recommended practices may vary between countries, these differences reflect the unique characteristics of each region, including variations in infrastructure and healthcare systems [5–9].

The mission of the CW campaign is to foster meaningful conversations between doctors and patients, guiding them towards evidence-based care that avoids unnecessary duplication of tests and procedures, minimizes harm, and addresses genuine patient needs [4]. However, as noted by Furlan et al. the campaign encompasses a broader vision, redefining healthcare and medical decision-making to adopt a mindset that respects not only patients but also economic and environmental resources [10]. Escalating health-related costs and shrinking budgets, particularly in low- and middle-income countries, are pushing patients, healthcare professionals, and services to reassess their practices and adopt the CW approach [11]. Despite the CW considerable progress in recent years, a key barrier to its full implementation remains the lack of awareness among healthcare professionals [12]. There is a limited amount of review literature on CW recommendations in oncology, highlighting the need for a comprehensive mapping of these recommendations globally [13, 14].

The Choosing Wisely initiative in oncology stands out from other medical fields due to the complexity and resource-intensive nature of cancer [11]. Oncology often involves high-cost interventions, such as targeted therapies, immunotherapies, and advanced diagnostics like genomic testing. These recommendations aim to balance cost and clinical benefit by discouraging treatments that provide minimal value or only marginally extend life [13]. What sets them apart is their strong emphasis on cost-conscious care, personalized treatment strategies, integration of prognosis and end-of-life considerations, multidisciplinary approaches, and efficient resource prioritization [10].

In addition to prioritizing clinical appropriateness and cost-effectiveness, the CW initiative aligns with the growing global imperative to address sustainability and planetary health, particularly relevant in oncology. As cancer care is highly resource-intensive, adopting waste-reductive strategies can serve dual purposes: enhancing healthcare efficiency and mitigating environmental impact. One illustrative approach is the redispensing of unused oral anticancer medications, which has demonstrated measurable benefits in terms of both cost savings and waste reduction. For instance, the ROAD study in the Netherlands showed a 68% reduction in drug waste, generating annual net savings of €576 to €1591 per patient, while ensuring medication safety through rigorous quality control measures [15]. Environmental evaluations of such practices also reported decreased pharmaceutical-related carbon emissions and potential ecological gains [16]. Similarly, another implementation study found that 79% of returned oral anticancer medication units met standardized quality criteria and were suitable for redispensing, resulting in approximately €483,000 in financial savings and a notable reduction in hazardous pharmaceutical waste over a single year [17]. Collectively, these findings illustrate the potential of CW principles to advance value-based oncology care that aligns with clinical efficacy, economic efficiency, and environmental sustainability. Integrating this dimension into the campaign further strengthens its relevance in an era of climate and resource challenges.

Thus, this study is aimed at mapping CW recommendations across various countries, creating a comprehensive guide to support healthcare providers, funders, and patients in adopting value-based care in oncology. The findings of this study will greatly benefit oncology by promoting evidence-based treatments and reducing resource waste. By synthesizing evidence on effective alternatives, fostering evidence-based discussions, and identifying research gaps, it will drive more informed decision-making and efficient care.

## Methods

### Protocol and registration

This scoping review was conducted following the Joanna Briggs Institute (JBI) methodology for scoping reviews [18] and reported in accordance with the Preferred Reporting Items for Systematic Reviews and Meta-Analyses extension for scoping reviews (PRISMA-ScR) [19]. A review protocol was developed to guide the process and registered with the Open Science Framework (10.17605/OSF.IO/FP23H).

### Eligibility criteria

The acronym PCC (Population, Concept, and Context) was used to formulate the question: “What are the current trends and available evidence regarding recommendations from the internationally recognized Choosing Wisely campaigns in oncology?” (P) not applicable; (C) Choosing Wisely recommendations; and (C) oncology.

We included reviews, observational, interventional studies, correspondence, reports, expert opinions, and editorials addressing CW recommendations associated with a recognized international CW campaign in oncology, from 2012 to 2024. There were no language restrictions. The exclusion criteria were as follows: (1) studies addressing CW recommendations associated with a recognized international CW campaign targeting the pediatric population; (2) studies addressing CW recommendation associated with a recognized international CW campaign targeting hematologic malignancy; (3) studies addressing value-based healthcare and not including the CW recommendation associated with a recognized international CW campaign; and (3) conference abstract.

Pediatric and hematologic tumors, while part of the broader field of oncology, do not align entirely with clinical oncology in the same way as solid tumors in adults, due to their distinct biological and clinical characteristics. As a result, they were not included in this research.

### Information sources and search

In collaboration with the research team, a search strategy was developed to identify CW recommendations from eligible citations published between January 1 st, 2012, and May 28, 2024, following the campaign’s launch in 2012. On May 28, 2024, searches were conducted in the following electronic databases: PubMed (via National Library of Medicine), Embase (via Elsevier), LILACS (via Virtual Health Library), and Web of Science Core Collection (WoSCC) (via Clarivate). The search strategy combined keywords and free-text terms, using Boolean operators AND/OR, and is detailed in Appendix 1.

A gray literature search was performed using Google Scholar and the ProQuest Dissertations & Theses Citation Index (via Clarivate). Additionally, a search for gray literature related to Choosing Wisely international campaigns was conducted using Google®. For each campaign website, we searched for recommendation lists using the terms “neoplasms,” “cancer,” and “oncology.” This approach ensured the inclusion of global literature, enhancing the comprehensiveness of the search.

The references were imported into the EndNote online version, where duplicates were removed. The remaining references were then exported to Rayyan® (Qatar Computing Research Institute, Qatar) for the selection phase, during which titles and abstracts were screened.

### Selection of sources of evidence

Initially, two independent reviewers (FVA and PEDR) screened all titles and abstracts based on predefined eligibility criteria. Subsequently, the same reviewers assessed the full-text articles of the citations identified during the initial screening, ensuring they met all inclusion criteria. Any discrepancies during the screening process were resolved through discussion. Finally, we reviewed the bibliographies and reference sections of the selected studies, applying the same inclusion criteria used in the previous screening.

### Data items and data charting process

Following the study selection process, data were collected by a primary reviewer (FVA) and verified by a second independent reviewer (PEDR). Recommendations were itemized and documented for each included study. Additionally, websites for international campaigns identified during the gray literature search were reviewed, and the recommendations from each campaign’s website were recorded. All data were organized and stored in Microsoft Excel® files to create tables of recommendations.

We then provided a comprehensive summary of all clinical recommendations, presented in tables and descriptive text. The recommendations were categorized according to various stages of the oncology care continuum, including screening and prevention, diagnosis and staging, treatment, palliative care, and surveillance. We noted the country or organization that endorsed each recommendation and the cancer location.

### Synthesis of results

A narrative summary was also drafted to synthesize the findings and describe the evidence relevant to the review’s objective.

## Results

### Selection of sources of evidence

**Fig. 1:**
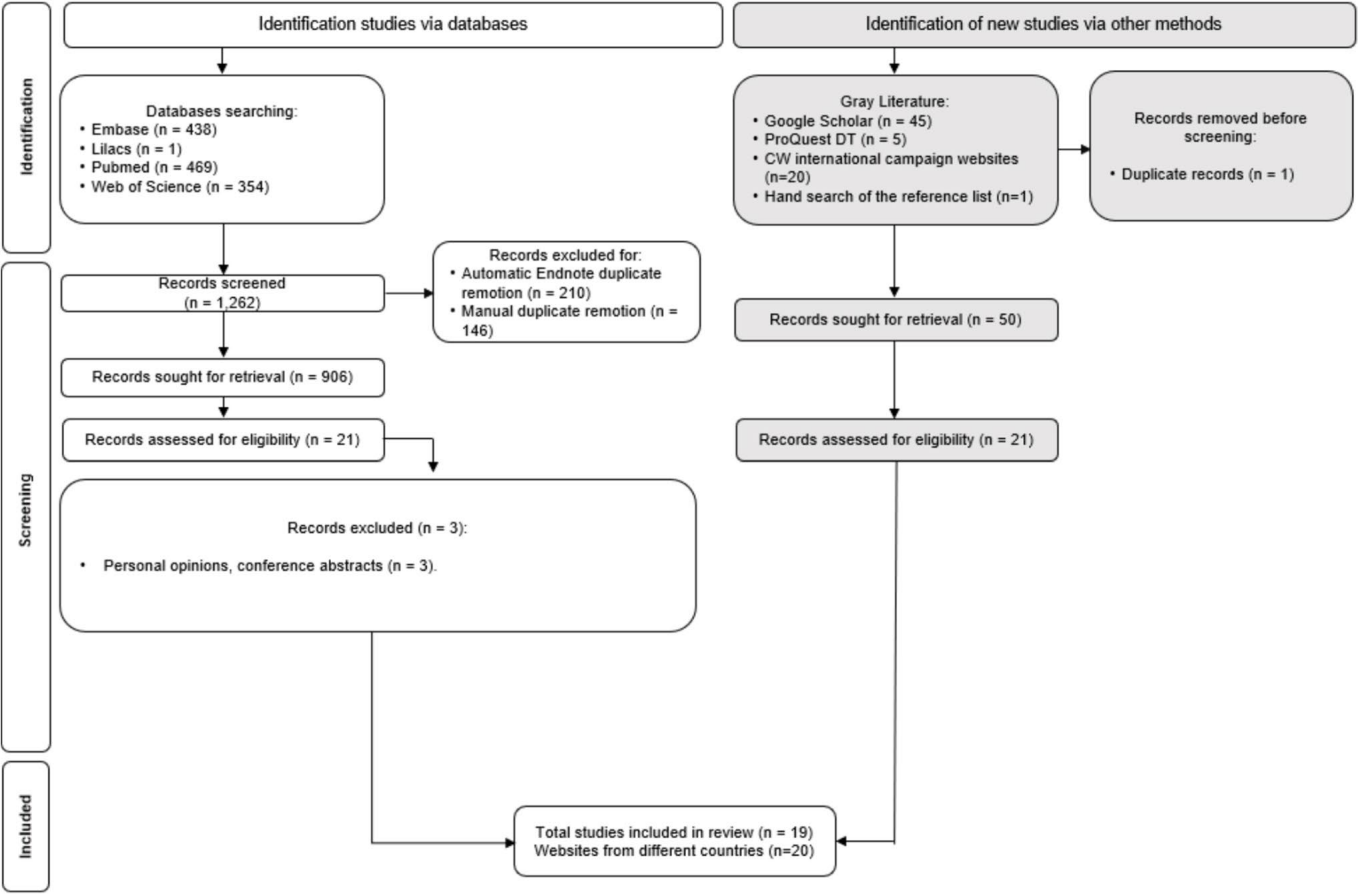
PRISMA 2020 flow diagram

From 1262 references identified through database searches, 906 remained after removing duplicates. These records were then assessed against eligibility criteria in two selection phases. In the first phase, titles and abstracts were read, leading to the selection of 21 studies for full-text review in the second phase. The gray literature search identified 1 record that was assessed for eligibility. Thereafter, 19 studies were finally included for descriptive synthesis. Studies that did not meet the eligibility criteria were excluded and are listed in Appendix 2. Finally, we searched for lists of Choosing Wisely recommendations related to recognized international oncology campaigns across 20 websites from gray literature, representing nine different countries (Appendix 3). Figure 1 illustrates the PRISMA 2020 flow diagram for eligible articles [20].

### Characteristics of sources of evidence

**Fig. 2:**
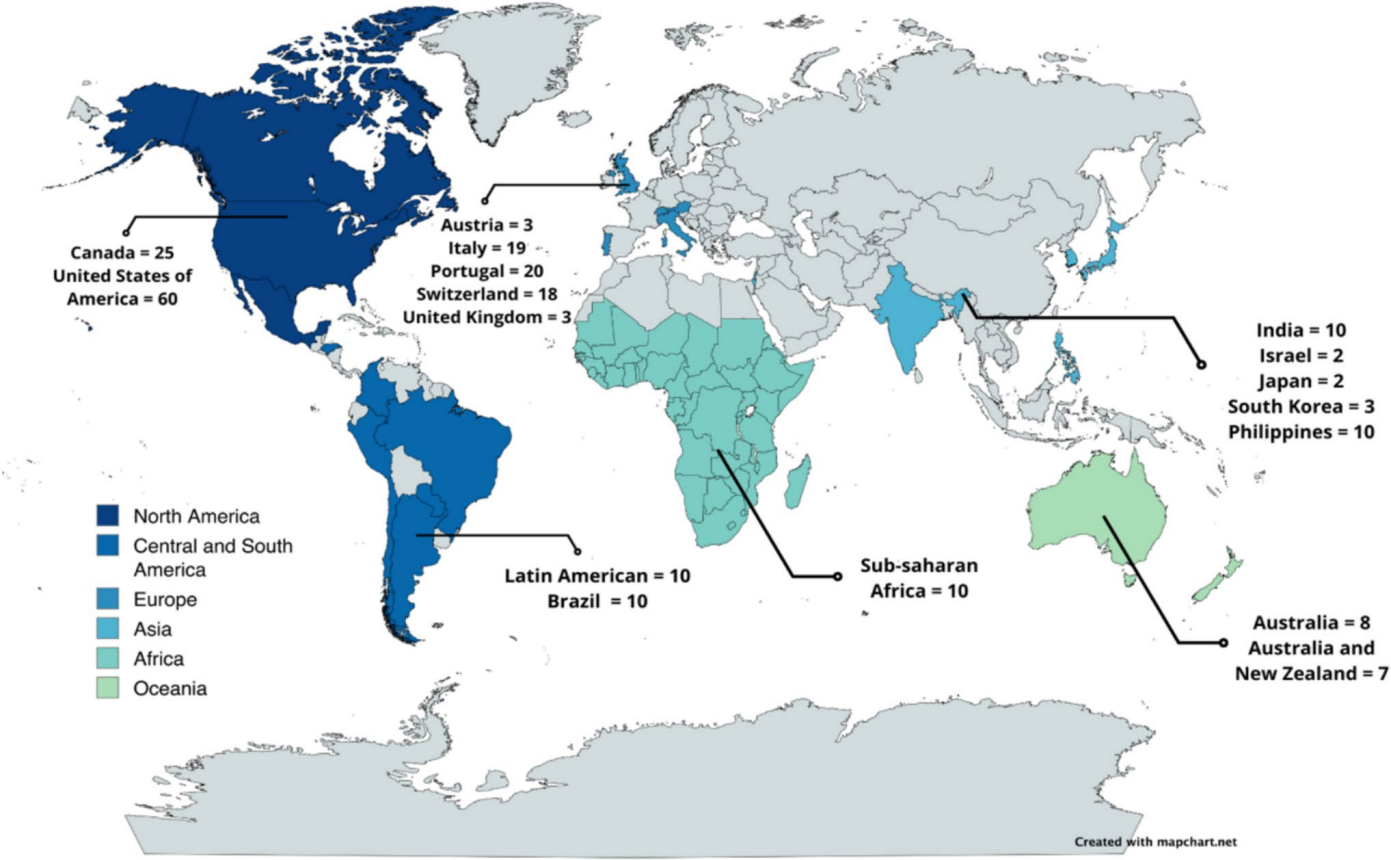
Worldwide distribution of Choosing Wisely recommendations in oncology (*n* = 220)

A total of 220 recommendations were gathered from 19 studies [6–9, 14, 21–34] and 20 websites recognized international CW campaigns [5, 35–53] in oncology. These recommendations are distributed worldwide, including North America [4, 9, 53], Latin America [8, 33], Asia [7, 30, 32, 34, 35], Europe [31, 48–52], Africa [6], and Oceania [47]. The specific distribution of recommendations across countries and continents is illustrated in Fig. 2.

**Fig. 3:**
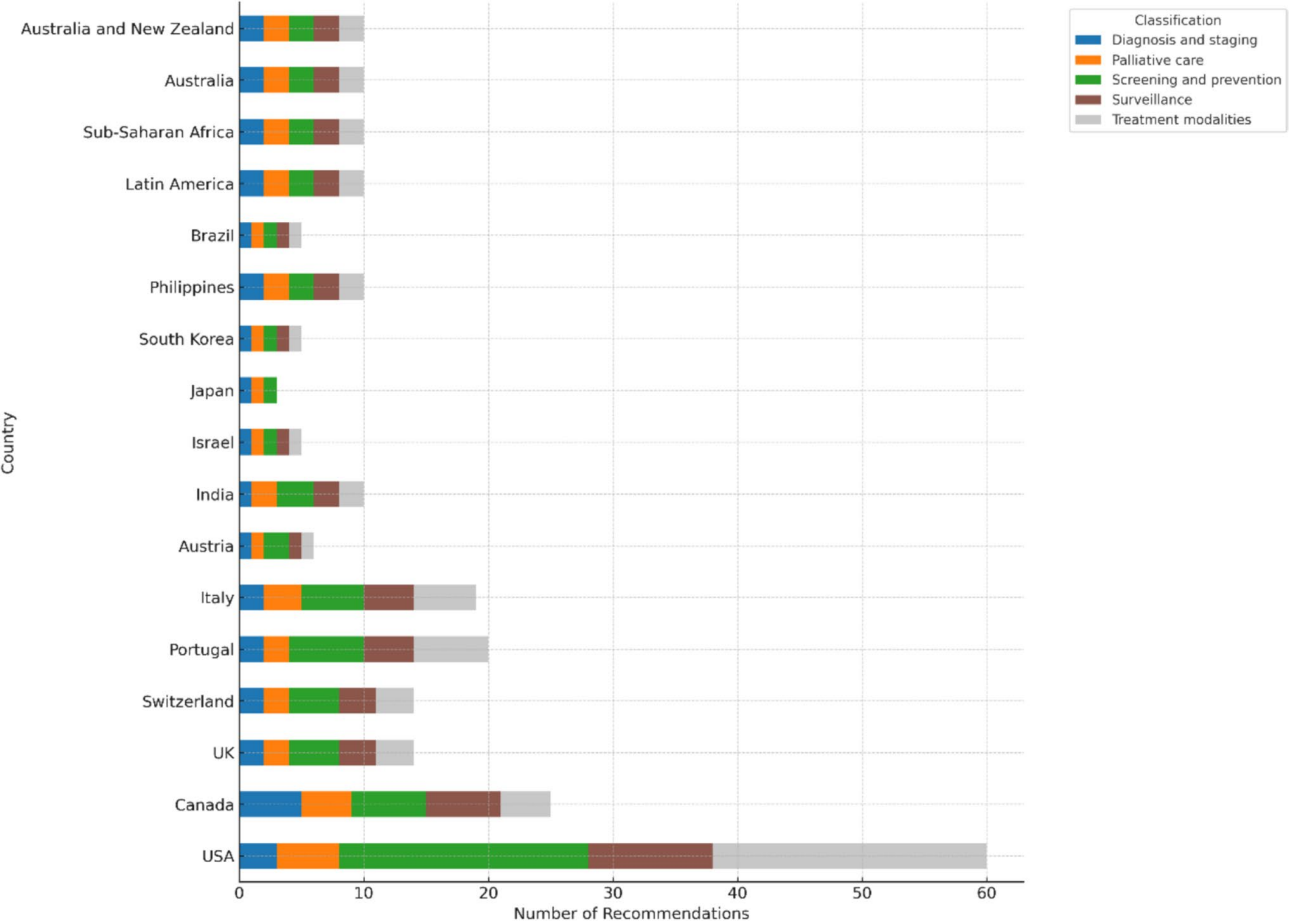
Distribution of Choosing Wisely recommendations by classification and countries and continents

The recommendations cover various aspects of oncology care, including screening and prevention for asymptomatic populations, diagnosis and staging of newly diagnosed patients, treatment modalities such as surgery, chemotherapy, and radiation therapy, palliative care, and surveillance after initial treatment. Figure 3 presents an integrated view of the geographic distribution and thematic classification of the recommendations, emphasizing cross-national and domain-specific variations in oncology care.

**Fig. 4:**
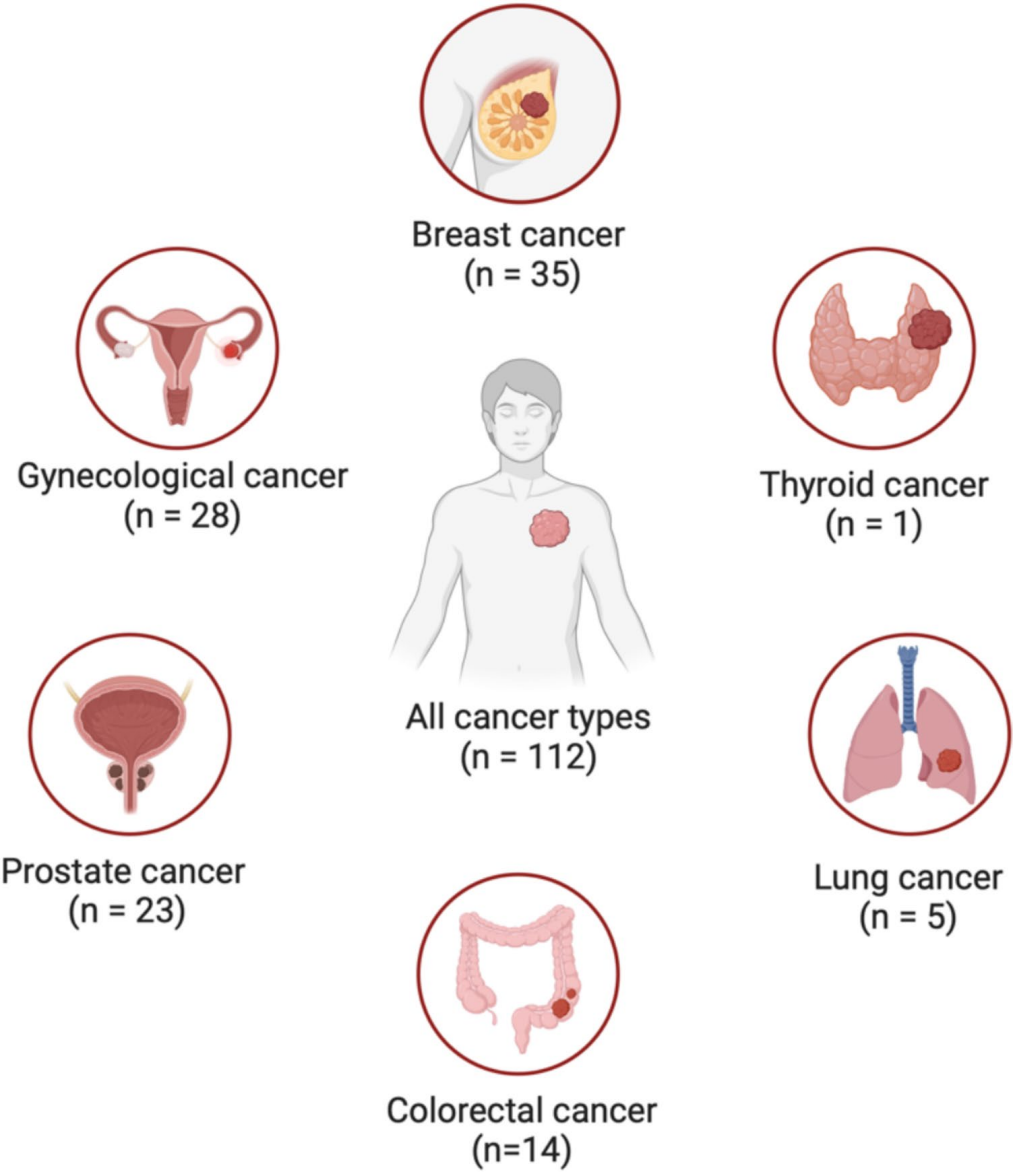
Distribution of Choosing Wisely recommendations by cancer type

Approximately half of the recommendations addressed all cancer types, while the remainder focused on specific tumors, including breast cancer, gynecological cancers (including cervical, ovarian, and endometrial cancers), prostate cancer, colorectal cancer, lung cancer, melanoma, and thyroid cancer. The detailed distribution of recommendations by cancer type is illustrated in Fig. 4.

FFIXThe complete checklist of CW recommendations is provided in Appendix 4, offering a structured overview organized by domain and cancer type, together with citations to the original sources from which these recommendations originate.

### Screening and prevention for asymptomatic populations

Many CW recommendations emphasize the importance of considering life expectancy before conducting cancer screenings. Multiple recommendations, particularly from specialty societies in the USA (such as the American Geriatrics Society and the Post-Acute and Long-Term Care Medical Association), as well as those from Canada, Portugal, and Australia, advise against screening for cancers like breast, colorectal, prostate, and lung when life expectancy is less than 10 years. This caution stems from concerns about the risks of overdiagnosis and overtreatment [8, 14, 26, 29, 32–34, 43, 46, 52].

Regarding breast cancer screening, recommendations specifically address mammography, advising against screening for women with a life expectancy of less than 5 years and recommending the initiation of routine annual mammograms at age 40 [44, 45, 49]. Additionally, there is a strong recommendation to avoid routine breast MRI for women at average risk [14, 44]. In terms of cervical cancer screening, recommendations from Canada, the USA and Portugal advise not to screen women over 65 with adequate prior screening or immunocompetent women under 21 years [23, 29, 38, 39, 53]. In addition, annual screening is no longer recommended [38, 39, 48, 49]. For prostate cancer screening, many recommendations advise against routine prostate cancer screening with a PSA or digital rectal examination without discussion risks and benefits with the patient [5, 23, 31, 35, 39, 47, 48, 50, 51].

There is a global consensus on avoiding the use of serum tumor markers and imaging techniques, such as PET-CT, for cancer screening in asymptomatic patients. All recommendations from Latin America, Japan, and the Philippines advise against the indiscriminate use of tumor markers for cancer screening in asymptomatic individuals or those at low to moderate risk. Similarly, 100% of recommendations from Japan, Italy, and Latin America recommend against PET-CT screening for asymptomatic adults and low-risk patients [8, 32–34, 52].

### Diagnosis and staging of newly diagnosed patients

Recommendations from the American Society of Clinical Oncology (ASCO), as well as from Canada, Portugal, and Italy, suggest limiting unnecessary imaging, such as PET-CT, for cancer staging—particularly in early-stage cancers with a low risk of metastasis [5, 14, 25, 28, 45, 49, 53]. Additionally, it is recommended to avoid using serum tumor markers for cancer diagnosis unless there is a clear clinical indication [14, 49, 52, 53].

### Treatment

Recommendations from India, Africa, and the Philippines stress the importance of involving multidisciplinary oncology teams in the decision-making process for potentially curable cancers to ensure that all aspects of care are considered, improving patient outcomes [6, 7, 32]. Additionally, recommendations from the USA, Switzerland, the Philippines, and India advise against using Granulocyte-Colony Stimulating Factors (G-CSF) unless the risk of febrile neutropenia is 20% or higher. This is a standardized threshold across these regions [5, 7, 32, 48].

Active surveillance for prostate cancer is recommended in several countries and continents, including the USA, Canada, Africa, and Latin America. It is advised not to initiate treatment for patients with low-risk prostate cancer (T1/T2, PSA < [10] ng/mL, Gleason score < 7) without first discussing the option of active surveillance [6, 9, 21, 22, 33, 47–49, 53]. Furthermore, the USA, Brazil, and Switzerland recommend against using molecular targeted therapies unless a predictive biomarker has been detected. This emphasizes the importance of enabling more precise and effective treatments tailored to each patient by considering the genetic and molecular characteristics of cancer, as well as the patient’s unique individual factors [5, 8, 48].

In radiation therapy, there is a notable shift toward recommending shorter courses of radiation, particularly in palliative care for bone metastases or in breast conservation therapy. Recommendations from the USA, Canada, Portugal, Brazil, Switzerland, Australia, and Africa advise using no more than a single fraction of palliative radiation for uncomplicated bone metastases [8, 9, 12, 21, 22, 40, 47–49, 54]. Additionally, recommendations from the USA, Canada, Switzerland, Portugal, Australia, India, and Africa suggest shorter radiation schedules in patients with early-stage invasive breast cancer or palliative settings [6, 7, 9, 14, 21, 22, 47, 48, 53].

Furthermore, stereotactic radiosurgery (SRS) is recommended over whole-brain radiation for patients with limited brain metastases and good performance status. Recommendations from Canada, Switzerland, and Australia advise against the use of whole-brain radiation in patients with limited brain metastases when SRS is available [9, 33, 47–49, 54].

### Palliative care

In patients with advanced cancer who have low performance status or are unlikely to benefit from further cancer-directed therapies, many countries recommend focusing on symptom management and prioritizing palliative care. The focus is on improving quality of life rather than aggressive treatment with little benefit [5–8, 14, 32, 33, 47, 48, 51, 52].

### Surveillance after initial treatment

Recommendations from Canada, the USA, and Latin America recommend avoiding routine imaging or biomarker testing for cancer recurrence in asymptomatic patients, particularly when such surveillance does not improve survival or quality of life. This approach helps minimize unnecessary interventions and reduce healthcare costs [5–7, 9, 14, 22, 23, 32, 33, 36, 37, 42, 44, 47, 51, 53]

## Discussion

The American Board of Internal Medicine (ABIM) launched the Choosing Wisely (CW) campaign to empower patients, healthcare providers, and specialty societies to make more informed and effective decisions regarding healthcare delivery [55]. In 2012, ASCO developed the Top Five list for oncology, highlighting tests and procedures that have limited evidence of benefit and should therefore be used minimally [56]. This issue is especially pertinent in oncology, where rising healthcare costs have prompted greater scrutiny of whether physicians are providing care that aligns with established guidelines [57, 58]. This scoping review has yielded a comprehensive list of Choosing Wisely recommendations for solid tumors. We conducted a thorough comparison of recommendations from various countries, analyzing both commonalities and differences. Our goal for this summary of current guidance is to form a platform that can guide practice and enhance the quality of oncologic care.

Across various societies, there is a clear emphasis on the careful selection of patients for screening, diagnostics, and treatments based on individual risk factors, including life expectancy and biomarker status [59–62]. The findings of this scoping review highlight a strong international consensus on avoiding unnecessary interventions in oncology, favoring more personalized, risk-based, and resource-efficient approaches to cancer care. Implementing CW recommendations in practice may encounter several challenges. Health system limitations, particularly in low- and middle-income countries, can restrict access to palliative care and symptom management due to resource constraints. Additionally, gaps in training and awareness among healthcare providers may result in inconsistent application of the latest guidelines. Cultural and social factors also could play a role; in some cultures, there is a stronger emphasis on aggressive treatments to combat the disease, often overshadowing the focus on comfort care [3, 12, 63]. The results of this scoping review have the potential to support the CW recommendations implementation by promoting knowledge dissemination among healthcare professionals.

Multidisciplinary care, which brings together a team of experts for decision-making in curative cancer treatments, is a prominent theme. This approach also applies to palliative care, focusing on resource optimization by prioritizing quality of life and steering clear of aggressive treatments for advanced cancer patients with poor performance status who are unlikely to benefit. This aligns with a broader strategy to avoid unnecessary diagnostic procedures that do not enhance outcomes and may instead lead to patient anxiety or unwarranted treatments [3]. Recommendations from low- and middle-income countries (LMICs), such as India, Africa, and the Philippines, emphasize the importance of involving multidisciplinary oncology teams in the treatment planning for curable cancers. Additionally, there is an increasing shift toward optimizing resources in cancer care by avoiding aggressive treatments for patients with poor prognosis, regardless of the country's income level.

Radiation therapy is a well-explored topic within the Choosing Wisely recommendations for cancer treatment. Both LMICs and high-income countries (HICs) address this topic; similarly, they aim to reduce the treatment burden on patients and to optimize resource utilization. For palliative radiation in uncomplicated bone metastases, recommendations advise limiting treatment to a single fraction. Regarding hypofractionation, recommendations suggest shorter radiation schedules for early-stage invasive breast cancer and in palliative settings. Additionally, all recommendations advise against whole-brain radiation for patients with limited brain metastases when stereotactic radiosurgery (SRS) is an option. This aligns with the trend of using more precise and less invasive treatment modalities that are equally effective [64–66].

Data from this scoping review reveals some differences in Choosing Wisely recommendations between LMICs and HICs. A key distinction is the emphasis on multidisciplinary care for curable cancers, a recommendation unique to LMICs [6, 7, 32]. Furthermore, discouraging the use of intensive care units unless a condition is reversible is mentioned in only one LMIC recommendation [7]. This may reflect a lack of comprehensive serious illness discussions in these regions, resulting in excessive and unnecessary interventions. At the same time, there are shared priorities across recommendations. Both LMICs and HICs stress the importance of personalizing screening, staging, and treatment based on individual risk factors, such as life expectancy and biomarker status. They also highlight the need to optimize palliative care and improve the efficient allocation of resources.

Value-based care delivery is essential for all health systems; however, it is particularly critical in LMICs due to their constrained health budgets and insufficient infrastructure for effective treatment delivery [67]. The rapid increase in cancer cases in these environments presents significant challenges, as there is often a shortage of trained professionals and sufficient healthcare capacity to manage this complex disease [68]. To address these issues, standard treatment guidelines, including resource-stratified guidelines for cancer care, are available to guide practice in LMICs [69–71]. These guidelines serve as a vital tool for promoting value-based care in these settings [12]. Developing or adapting CW recommendations into resource-stratified guidelines offers a promising strategy to enhance CW implementation, particularly in LMICs. This approach aligns with capacity-building efforts, ensuring that CW initiatives are contextually relevant and practically applicable within resource-limited healthcare systems.

While this scoping review provides a comprehensive overview of CW recommendations across diverse global contexts, it does not evaluate which measures have demonstrated greater effectiveness in clinical practice, nor does it systematically explore the barriers that may hinder their implementation. Recent studies have shown that the success of specific recommendations often depends on local healthcare system capacity, provider engagement, and sociocultural values. For example, a systematic review demonstrated that active, multi-component interventions targeting clinician behavior are significantly more effective in reducing low-value care than passive dissemination strategies [2]. Qualitative research conducted in Canadian hospitals further identified key contextual factors influencing implementation, such as institutional leadership, availability of performance data, and clarity of local targets [72].

Additionally, the Choosing Wisely De-implementation Framework (CWDIF) highlights the importance of adapting recommendations to the realities of clinical workflow, health system constraints, and local values in order to achieve sustainable adoption [73]. These findings underscore the need for a deeper understanding of how contextual dynamics influence the uptake and impact of CW recommendations. Future research should prioritize comparative implementation studies across settings, aiming to identify facilitators and barriers to adoption and develop strategies to tailor CW guidance to diverse healthcare environments, ultimately supporting their global applicability and sustainability.

The limitations of our scoping review may include the potential inadvertent eligibility of studies. Since our search strategy focused narrowly on the CW initiative, we acknowledge that this scope may have led to the exclusion of relevant initiatives that align with CW principles but are not branded under the Choosing Wisely label. This includes regional or institutional efforts promoting sustainable or low-value care reduction, particularly in contexts where the CW branding is less prevalent. This focused strategy was deliberate, as the objective was to map the recommendations that explicitly stem from the CW initiative to ensure comparability and traceability across countries. While this may limit the inclusion of broader sustainable oncology practices, the decision was justified by the aim of documenting the global implementation of the CW framework specifically. We recognize that future reviews may complement this work by incorporating sustainability-focused campaigns or frameworks beyond CW.

## Conclusion

In summary, this review suggests a global movement toward more rational and patient-centered oncology care, minimizing unnecessary interventions and maximizing the benefit of personalized, evidence-based treatments. In addition, involving a multi-disciplinary team of experts for decision-making in curative cancer treatments is critical for patient outcomes. Furthermore, there is a growing shift toward optimizing resources in cancer care by focusing on quality of life and avoiding aggressive treatments in advanced cancer patients with poor performance status and prognosis.

Rather than evaluating implementation or outcomes, this review provides a descriptive synthesis of CW recommendations across countries and domains of oncology care. As such, the findings offer a foundation for future investigations into how these recommendations are developed, interpreted, and potentially adopted in different healthcare contexts. One potential area for further exploration is how CW guidance might be adapted into resource-stratified frameworks, particularly in LMICs, to ensure alignment with local healthcare capacities and clinical priorities.

## Supplementary Information

Below is the link to the electronic supplementary material.

ESM 1 DOCX (883 KB)

ESM 2 DOCX (22.4 KB)

ESM 3 DOCX (37.2 KB)

ESM 4 DOCX (1.02 MB)

## Data Availability

No datasets were generated or analysed during the current study.
