# Supplementary material for: Choosing Wisely recommendations in oncology: a scoping review
Source: Support Care Cancer. 2026 Mar 4;34(3):276. doi: 10.1007/s00520-026-10437-z (PMC12960452; doi:10.1007/s00520-026-10437-z)
Supplement: Supplementary file 1 — DOCX (883 KB) [file 520_2026_10437_MOESM1_ESM.docx]

**Choosing Wisely recommendations in oncology: a scoping review**

Appendix 1. Search Strategy performed in databases Pubmed, Embase, LILACS, Web of Science Core Collection, Google Scholar, and Proquest Thesis and Dissertations on May 28^th^, 2024.

| Database | Search strategy | Result |
| --- | --- | --- |
| PubMed | ("value based health care"[MeSH Terms] OR "value based health care"[All Fields] OR ("choos*"[All Fields] AND "wise*"[All Fields]) OR "choosing wisely"[All Fields]) AND ("medical oncology"[MeSH Terms] OR "medical oncology"[All Fields] OR "cancer"[All Fields] OR "cancers"[All Fields] OR "cancer s"[All Fields] OR "cancerated"[All Fields] OR "canceration"[All Fields] OR "cancerization"[All Fields] OR "cancerized"[All Fields] OR "cancerous"[All Fields] OR "neoplasms"[MeSH Terms] OR "neoplasms"[All Fields] OR "neoplasm s"[All Fields] OR "neoplasm"[All Fields] OR "oncology"[All Fields] OR "oncology s"[All Fields]) | 469 |
| Embase | ("value based health care" OR choos* wise* OR "choosing wisely") AND ("medical oncology" OR "cancer" OR "cancers" OR "cancer s" OR "cancerated" OR "canceration" OR "cancerization" OR "cancerized" OR "cancerous" OR "neoplasms" OR "neoplasms" OR "neoplasm s" OR "neoplasm" OR "oncology" OR "oncology s") AND [embase]/lim NOT ([embase]/lim AND [medline]/lim) | 438 |
| LILACS | ("value based health care" OR “Cuidados de Saúde Baseados em Valores” OR “Atención Médica Basada en Valor” OR “Soins de santé basés sur la valeur” OR "choosing wisely") AND ("neoplasms" OR "neoplasias" OR "tumeurs" OR "cancer" OR "câncer" OR "cáncer") | 1 |
| Web of Science Core Collection (WoSCC) | TS=("value based health care" OR "choosing wisely") AND TS=("medical oncology" OR "cancer" OR "cancers" OR "cancer s" OR "cancerated" OR "canceration" OR "cancerization" OR "cancerized" OR "cancerous" OR "neoplasms" OR "neoplasms" OR "neoplasm s" OR "neoplasm" OR "oncology" OR "oncology s") | 354 |
| Google Scholar | allintitle: ("value based health care" OR "choosing wisely") AND ("medical oncology" OR "cancer" OR "cancers" OR "cancer s" OR "cancerated" OR "canceration" OR "cancerization" OR "cancerized" OR "cancerous" OR "neoplasms" OR "neoplasms" OR "neoplasm s" OR "neoplasm" OR "oncology" OR "oncology s") | 45 |
| ProQuest Thesis and Dissertations | TS=("value based health care" OR "choosing wisely") AND TS=("medical oncology" OR "cancer" OR "cancers" OR "cancer s" OR "cancerated" OR "canceration" OR "cancerization" OR "cancerized" OR "cancerous" OR "neoplasms" OR "neoplasms" OR "neoplasm s" OR "neoplasm" OR "oncology" OR "oncology s") | 5 |
