# Supplementary material for: Choosing Wisely recommendations in oncology: a scoping review
Source: Support Care Cancer. 2026 Mar 4;34(3):276. doi: 10.1007/s00520-026-10437-z (PMC12960452; doi:10.1007/s00520-026-10437-z)
Supplement: Supplementary file 2 — DOCX (22.4 KB) [file 520_2026_10437_MOESM2_ESM.docx]

**Choosing Wisely recommendations in oncology: a scoping review**

Appendix 2. Excluded studies

| Title | Year | Journal | Authors | Exclusion reason |
| --- | --- | --- | --- | --- |
| Choosing Wisely Africa; 10 low-value or harmful practices that should be avoided in cancer care, | 2020 | Journal of Clinical Oncology | Fidel Rubagumya, Gunita Mitera, Sidy Ka et al. | Abstract |
| Choosing Wisely recommendations compliance in bone scan scintigraphies of newly diagnosed prostate cancer patients | 2020 | European Journal of Nuclear Medicine and Molecular Imaging | Saura Lopez, Á. O. Rabines Juárez, N. A. Rudic Chipe, M. J. et al | Abstract |
| Choosing Wisely | 2014 | Practical Radiation Oncology | Colleen Lawton | Personal opinion |
