# Supplementary material for: Choosing Wisely recommendations in oncology: a scoping review
Source: Support Care Cancer. 2026 Mar 4;34(3):276. doi: 10.1007/s00520-026-10437-z (PMC12960452; doi:10.1007/s00520-026-10437-z)
Supplement: Supplementary file 3 — DOCX (37.2 KB) [file 520_2026_10437_MOESM3_ESM.docx]

**Choosing Wisely recommendations in oncology: a scoping review**

Appendix 3. List of Choosing Wisely websites from grey literature seach

| **ID** | **Website** | **Accessed** | **Country** |
| --- | --- | --- | --- |
| 1 | <https://choosingwiselyitaly.org/en/raccomandazioni/> | 19 July 2024 | Italy |
| 2 | <https://choosingwiselycanada.org/recommendations/> | 19 July 2024 | Canada |
| 3 | <https://choosingwisely.co.uk/recommendations-archive/> | 20 July 2024 | United Kingdom |
| 4 | <https://www.gemeinsam-gut-entscheidn.at/> | 20 July 2024 | Austria |
| 5 | <https://ordemdosmedicos.pt/choosing-wisely-portugal-escolhas-criteriosas-em-saude> | 20 July 2024 | Portugal |
| 6 | [https://www.smartermedicine.ch/de/top-5-listen/radio-onkol ogie.html](https://www.smartermedicine.ch/de/top-5-listen/radio-onkol%20ogie.html) | 20 July 2024 | Switzerland |
| 7 | <https://www.choosingwisely.org.au/recommendations> | 30 July 2024 | Australia |
| 8 | <https://society.asco.org/news-initiatives/current-initiatives/cancer-care-initiatives/value-cancer-care/choosing-wisely> | 30 July 2024 | USA |
| 9 | <https://paltc.org/programs/choosing-wisely> | 30 July 2024 | USA |
| 10 | <https://www.breastsurgeons.org/resources/choosing_wisely> | 30 July 2024 | USA |
| 11 | <https://www.surgonc.org/wp-content/uploads/2020/11/SSO-5things-List_2020-Updates-11-2020.pdf> | 30 July 2024 | USA |
| 12 | <https://www.healthinaging.org/choosing-wisely> | 30 July 2024 | USA |
| 13 | <https://www.facs.org/media/wegncq3f/coclist.pdf> | 30 July 2024 | USA |
| 14 | <https://www.facs.org/media/xp3msgu0/acslist.pdf> | 30 July 2024 | USA |
| 15 | <https://aahpm.org/outreach/choosing-wisely> | 30 July 2024 | USA |
| 16 | <https://www.aafp.org/family-physician/patient-care/clinical-recommendations/choosing-wisely.html> | 30 July 2024 | USA |
| 17 | <https://www.acog.org/practice-management/patient-safety-and-quality/partnerships/choosing-wisely> | 30 July 2024 | USA |
| 17 | <https://www.sgo.org/wp-content/uploads/2023/12/Choosing-Wisely-Five-Tips-for-a-Meaningful-Conversation-Between-Patients-and-Providers.pdf> | 30 July 2024 | USA |
| 19 | <https://choosingwiselyitaly.org/en/societa/aimn-2/> | 1 August 2024 | Italy |
| 20 | <https://www.ima.org.il/MedicineQuality/ChoosingWisely.aspx> | 1 August 2024 | Israel |
