# Supplementary material for: Choosing Wisely recommendations in oncology: a scoping review
Source: Support Care Cancer. 2026 Mar 4;34(3):276. doi: 10.1007/s00520-026-10437-z (PMC12960452; doi:10.1007/s00520-026-10437-z)
Supplement: Supplementary file 4 — DOCX (1.02 MB) [file 520_2026_10437_MOESM4_ESM.docx]

**Choosing Wisely recommendations in oncology: a scoping review**

Appendix 3. Summary of all CW clinical recommendations

| **ID** | **Recommendation** | **Condition** | **Country/organizations supporting this recommendation** |
| --- | --- | --- | --- |
| **SCREENING AND PREVENTION (n=60)** | | | |
| 1 | Do not recommend screening for breast, colorectal, prostate or lung cancer without considering life expectancy and the risks of testing, over-diagnosis and overtreatment^1^. | Cancer | USA / AGS, AMDA, SGIM |
| 2 | Don’t recommend screening for breast, colorectal or prostate cancer if life expectancy is estimated to be less than 10 years^2,3^. | Cancer | USA / PALTmed |
| 3 | Don’t recommend screening for breast or colorectal cancer, nor prostate cancer (with the PSA test) without considering life expectancy and the risks of testing, overdiagnosis and overtreatment^4^. | Cancer | USA / AGS |
| 4 | Do not use serum tumour markers indiscriminately for the screening and diagnosis of cancer^5^. | Cancer | Philippinnes |
| 5 | Do not perform screening diagnostic tests such as ultrasound, positron emission tomography/computerized tomography, or serum tumor marker testing in asymptomatic patients or those with a low to moderate risk^6^. | Cancer | Brazil |
| 6 | Do not test for tumour markers except to assess or monitor active disease^+7^. | Cancer | Latin America |
| 7 | Do not recommend PET-CT cancer screening for asymptomatic adults^8^. | Cancer | Japan |
| 8 | Do not use 18F-FDG (Fluorodeoxyglucose) PET-CT as a "screening" test for cancer in healthy subjects^9,10^. | Cancer | Italy |
| 9 | Do not recommend tumor marker screening for asymptomatic adults^8^. | Cancer | Japan |
| 10 | Do not perform PET (positron emission tomography) or CT (computed tomography) for cancer screening in healthy subjects^7^. | Cancer | Latin America |
| 11 | Do not perform routine cancer screening, or surveillance for a new primary cancer, in the majority of patients with metastatic disease^11-13^. | Cancer | Canada |
| 12 | Do not perform routine cancer screening, or surveillance for a new primary cancer, in the majority of patients with metastatic disease^14^. | Cancer | Australia |
| 13 | Do not encourage the extensive and indiscriminate use of vitamin and mineral supplements as preventive elements for cancer and cardiovascular disease^9^. | Cancer | Italy |
| 14 | Do not neglect to address lifestyle factors such as smoking cessation and exercise throughout the patient’s cancer journey^6^. | Cancer | Brazil |
| 15 | Do not order screening or routine chronic disease testing such as thyroid-stimulating hormone, T3 and T4 for thyroid, hemoglobin A1C for diabetes or hemoglobin levels for anemia simply because a blood draw is taken^6.^ | Cancer | Brazil |
| 16 | Do not perform screening mammography in asymptomatic patients with normal exams who have less than 5-year life expectancy^15^. | Breast cancer | USA / ASBrS |
| 17 | Choose not to request screening mammograms for women with a life expectancy of less than 5 years^16^. | Breast cancer | Portugal |
| 18 | Do not routinely use breast MRI for breast cancer screening in average-risk women^3,17^. | Breast cancer | USA / SSO |
| 19 | Choose not to substitute screening mammography for breast ultrasound^3,16^. | Breast cancer | Portugal |
| 20 | Choose not to delay breast cancer screening until 50; choose to start at 40 on an annual basis^16^. | Breast cancer | Portugal |
| 21 | Choose not to request breast ultrasound as a complement to mammographic screening for breast cancer in women without dense breasts and who are not at high risk^16^. | Breast cancer | Portugal |
| 22 | Do not screen women older than 65 years of age for cervical cancer who have had adequate prior screening and are not otherwise at high risk for cervical cancer^18^. | Cervical cancer | USA / AAFP |
| 23 | Don’t perform screening for cervical cancer in low-risk women aged 65 years or older^19^. | Cervical cancer | USA / ACPM |
| 24 | Do not routinely screen with Pap smears if under 21 years of age or over 69 years of age^11^. | Cervical cancer | Canada |
| 25 | Do not perform cervical cytology (Pap test) or HPV screening in immunocompetent women under age 21^1^. | Cervical cancer | USA / ASCCP,  AAFP, ACPM |
| 26 | Do not perform pelvic exams on asymptomatic nonpregnant women, unless necessary for guideline appropriate screening for cervical cancer^18^. | Cervical cancer | USA / AAFP |
| 27 | Do not screen women younger than 30 years of age for cervical cancer with HPV testing, alone or in combination with cytology, or order screening tests for low-risk HPV types^18^. | Cervical cancer | USA / AAFP |
| 28 | Do not perform vaginal cytology (Pap test) or HPV screening in women who had hysterectomy (with removal of the cervix) for reasons other than high-grade cervical dysplasia (CIN 2/3) or cancer^1^. | Cervical cancer | USA/ ASCCP, AAFP, ACPM |
| 29 | Choose not to perform cervical-vaginal cytology (Pap test) or HPV screening in women who have already undergone hysterectomy (or removal of the cervix - trachelectomy), for reasons other than high-grade intraepithelial squamous lesion (HSIL, CIN 2 or 3) or carcinoma^16^. | Cervical cancer | Portugal |
| 30 | Don't perform Pap smears on women under the age of 21 or women who have had a hysterectomy for non-cancer disease^18^. | Cervical cancer | USA / AAFP |
| 31 | Do not perform annual cervical cytology (Pap test) or annual HPV screening of immunocompetent women with a history of negative screening^20^. | Cervical cancer | USA / ACOG |
| 32 | Don’t perform routine annual cervical cytology screening (Pap tests) in women 30–65 years of age^20^. | Cervical cancer | USA / ACOG |
| 33 | Choose not to perform cervical cytology (Pap smear) annually on women between 25 and 65 years of age^16^. | Cervical cancer | Portugal |
| 34 | No annual cytological smear as part of regular gynecological check-ups^21^. | Cervical cancer | Switzerland |
| 35 | Don’t screen low risk women with CA-125 or ultrasound for ovarian cancer^22^. | Ovarian cancer | USA / SGO |
| 36 | Do not screen for ovarian cancer in asymptomatic women at average risk^20^. | Ovarian cancer | USA / ACOG |
| 37 | Do not screen for ovarian cancer in asymptomatic women at avarage risk^11^. | Ovarian cancer | Canada |
| 38 | Do not perform pelvic ultrasound in average-risk women to screen for ovarian cancer^20^. | Ovarian cancer | USA / ACOG |
| 39 | Routine screening for ovarian cancer is not recommended for women without an increased risk. The potencial harm outweighs the benefit^23^. | Ovarian cancer | Austria |
| 40 | Do not routinely perform PSA-based screening for prostate cancer^19^. | Prostate cancer | USA / ACPM |
| 41 | Do not routinely perform PSA-based screening for prostate cancer^23^. | Prostate cancer | Austria |
| 42 | Unless a patient is at increased risk of prostate cancer because of race or family history, PSA testing does not necessarily lead to a longer life^24^. | Prostate cancer | United Kingdom |
| 43 | Before undergoing prostate screening tests, men should be informed about their individual risk and possible damage^23,25^. | Prostate cancer | Austria |
| 44 | Avoid PSA testing to detect prostate cancer without discussing the risks and benefits with the patient^21^. | Prostate cancer | Switzerland |
| 45 | Do not routinely screen for prostate cancer using a prostate-specific antigen (PSA) test or digital rectal exam. For men who desire PSA screening, it should only be performed after engaging in shared decision making^18^. | Prostate cancer | USA / AAFP |
| 46 | Screening for prostate cancer using a rectal exam or a PSA test is not recommended without a prior discussion between the patient and the doctor. If the patient requests a test, a full explanation must be given before it is performed, about the meaning of the expected results and their consequences^26^. | Prostate cancer | Israel |
| 47 | Don’t routinely perform PSA testing for prostate cancer screening in men with no symptoms of the disease^27^. | Prostate cancer | USA / ASCO |
| 48 | Do not perform PSA testing for prostate cancer screening in men with no symptoms and whose life expectancy is less than 7 years^14^. | Prostate cancer | Australia |
| 49 | Do not repeat colorectal cancer screening (by any method) in average-risk individuals for 10 years after a high-quality colonoscopy that does not detect neoplasia^1^. | Colorectal cancer | USA / AGA |
| 50 | Colorectal cancer screening (whatever the method) in individuals at average risk should be repeated no earlier than ten years after a negative total colonoscopy^21^. | Colorectal cancer | Switzerland |
| 51 | Avoid colorectal cancer screening tests on asymptomatic patients with a life expectancy of less than 10 years and no family or personal history of colorectal neoplasia^28^. | Colorectal cancer | USA / ACS |
| 52 | Avoid colorectal cancer screening tests in asymptomatic patients with a life expectancy of less than 10 years and with no personal or family history of colorectal neoplasia^11^. | Colorectal cancer | Canada |
| 53 | Choose not to recommend colorectal cancer (CRC) screening in users who are asymptomatic, have no family history, and have a life expectancy of <10 years^16^. | Colorectal cancer | Portugal |
| 54 | Avoid colorectal cancer screening tests in asymptomatic patients with a life expectancy of less than ten years without a family or personal history of colorectal neoplasia^21^. | Colorectal cancer | Switzerland |
| 55 | Don’t order colonoscopy as a screening test for bowel cancer in people at average or slightly above average risk. Use faecal occult blood screening instead^14^. | Colorectal cancer | Australia |
| 56 | Prescribe fecal occult blood test (FOBT) only for screening of colorectal cancer^9^. | Colorectal cancer | Italy |
| 57 | There is no need to perform a fecal occult blood test for 5 years after a normal, good quality colonoscopy^26^. | Colorectal cancer | Israel |
| 58 | Choose not to perform a Fecal Occult Blood Test (PSOF) within 5 years of a normal screening colonoscopy with adequate preparation^16^. | Colorectal cancer | Portugal |
| 59 | Do not perform CT scan screening for lung cancer among patients at low risk for lung cancer^29,30^. | Lung cancer | USA / ATS |
| 60 | Do not perform CT screening for lung cancer among patients at low risk for lung cancer^11^. | Lung cancer | Canada |
|  |  |  |  |
| **DIAGNOSIS AND STAGING (n=15)** | | | |
| 1 | Do not do unnecessary imaging for cancer staging, treatment planning, and image verification in the context of clinical yield. Imaging is carbon intensive^11^. | Cancer | Canada |
| 2 | Choose not to determine tumor biomarkers in the initial diagnostic approach^16^. | Cancer | Portugal |
| 3 | Don’t routinely prescribe serum cancer markers during the diagnostic or staging procedures in cancer care^9^. | Cancer | Italy |
| 4 | Do not ask for the determination of serum biomarkers like CEA, CA-125, HE4, CA-15.3, a-fetoprotein or CA-19.9 for the diagnosis of neoplastic disease in asymptomatic individuals^9^. | Cancer | Italy |
| 5 | Limit the use of diagnostic procedures in case of low-risk prostate and breast cancer. Avoid the use of serum biomarkers in early-stage breast cance^9^. | Cancer | Italy |
| 6 | Do not routinely order breast magnetic resonance imaging in new breast cancer patients with average risk^3,15,31,32^. | Breast cancer | USA / ASBrS |
| 7 | Don’t perform PET, CT, and radionuclide bone scans in the staging of early breast cancer at low risk for metastasis^3,27,32^. | Breast cancer | USA / ASCO |
| 8 | Choose not to request X-rays, Abdomino-pelvic Ultrasounds, Computed Tomography (CT) or Positron Emission Tomography (PET) or Bone Scans when staging early breast cancer with a low risk of metastasis^16^. | Breast cancer | Portugal |
| 9 | Do not prescribe serum tumor markers or genomic testing in unselected patients with breast cancer^3,11^. | Breast cancer | Canada |
| 10 | Do not routinely order specialized tumor gene testing in all new breast cancer patients^15,31,32^. | Breast cancer | USA / ASBrS |
| 11 | Don’t perform PET, CT, and radionuclide bone scans, or newer imaging scans in the staging of early prostate cancer at low risk for metastasis^27^. | Prostate cancer | USA / ASCO |
| 12 | Do not do routine bone scans in men with low-risk prostate cancer^11^. | Prostate cancer | Canada |
| 13 | Do not order a routine bone scan and CT scan of the pelvis in men with low-risk prostate cancer^11^. | Prostate cancer | Canada |
| 14 | Don’t perform lymphoscintigraphy and radioguided biopsy of the sentinel node in patients affected by cutaneous melanoma thinner than 0.75 mm, with no ulceration and mitotic rate <1/mm2^10^. | Melanoma | Italy |
| 15 | Do not perform an open biopsy or excision of a neck mass without having first considered a fine needle aspiration (FNA) biopsy^33^. | Thyroid cancer | Canada |
|  |  |  |  |
| **TREATMENT (n=93)** | | | |
| **Treatment– General (n=27)** | | | |
| 1 | Do not decide treatment for potentially curable cancers without inputs from a multidisciplinary oncology team^34^. | Cancer | India |
| 2 | Do not decide treatment of potentially curable cancers without inputs from a multidisciplinary oncology team^35^. | Cancer | Africa |
| 3 | Do not decide treatment for potentially curable cancers without inputs from a multidisciplinary oncology team^5^. | Cancer | Philippinnes |
| 4 | Do not treat patients with advanced metastatic cancer in the intensive care unit unless there is an acutely reversible event^34^. | Cancer | India |
| 5 | Do not forget to discuss about alternative/herbal medications including its potential harmful consequences while on active cancer treatment^5^. | Cancer | Philippinnes |
| 6 | Do not routinely offer pharmacological venous thromboembolism (VTE) prophylaxis to ambulatory outpatients who are undergoing oncological treatment^14^. | Cancer | Australia |
| 7 | Don’t use prophylactic white cell stimulating factors unless the expected risk of febrile neutropenia associated with a chemotherapy agent or regimen is equal to or greater than 20%^27^. | Cancer | USA / ASCO |
| 8 | Do not use white cell stimulating factors for primary prevention of febrile neutropenia for patients with less than 20% risk for this complication^34^. | Cancer | India |
| 9 | Avoid the use of granulocyte-colony stimulating factor (G-CSF) for primary prevention of febrile neutropenia for patients with less than 10%–20% risk for this complication^5^. | Cancer | Philippinnes |
| 10 | No prophylactic administration of granulocyte-stimulating growth factors (G-CSF) after chemotherapy unless the risk of febrile neutropenia is at least 20%^21^. | Cancer | Switzerland |
| 11 | Hyperthermia as a single treatment is not recommended for treating cancer^36^. | Cancer | Korea |
| 12 | Don’t initiate cancer treatment without defining the extent of the cancer (through clinical staging) and discussing with the patient the intent of treatment^37^. | Cancer | USA / CC |
| 13 | Do not initiate cancer treatment without defining the extent of the cancer (through clinical staging) and discussing the intent of treatment with the patient^35^. | Cancer | Africa |
| 14 | Do not initiate cancer treatment without confirming the diagnosis, defining the extent of the cancer (through clinical staging) and discussing the intent of treatment with the patient^5^. | Cancer | Philippinnes |
| 15 | Do not start cancer treatment without defining the extent of the disease (staging) and discussing with the patient the intention of treatment^7^. | Cancer | Latin America |
| 16 | Don’t give patients starting on a chemotherapy regimen that has a low or moderate risk of causing nausea and vomiting antiemetic drugs intended for use with a regimen that has a high risk of causing nausea and vomiting^27^. | Cancer | USA / ASCO |
| 17 | No prophylactic administration of antiemetics indicated primarily in moderately or strongly emetogenic chemotherapies, if the chemotherapy has low or no emetogenic potential^21^. | Cancer | Switzerland |
| 18 | Do not initiate management of low-risk prostate cancer without discussing active surveillance^38^. | Prostate cancer | USA / ASTRO |
| 19 | Do not treat low-risk clinically localized prostate cancer (eg, Gleason score < 7, PSA < 10.0 ng/mL, and tumor stage ≤ T2) without discussing active surveillance as part of the shared decision-making process^35^. | Prostate cancer | Africa |
| 20 | Don’t initiate management in patients with low-risk prostate cancer (T1/T2, PSA < 10 ng/ml, and Gleason score < 7) without first discussing active surveillance^11-13^. | Prostate cancer | Canada |
| 21 | Do not treat low-risk clinically localized prostate cancer where the Gleason score is < 7, PSA < 10.0 ng ml-1 or tumor stage ≤ T2 without discussing active surveillance as part of the shared decision-making process^11^. | Prostate cancer | Brazil |
| 22 | Do not initiate management in patients with low-risk prostate cancer (T1/T2, PSA < 10 ng/ml, and Gleason score < 7) without first discussing active surveillance^7^. | Prostate cancer | Latin America |
| 23 | Choose not to initiate treatments in patients with low-risk prostate carcinoma without considering active surveillance^16^. | Prostate cancer | Portugal |
| 24 | Do not begin treatment of low-risk prostate cancer without considering active surveillance(21). | Prostate cancer | Switzerland |
| 25 | Don’t initiate management of low-risk prostate cancer without discussing active surveillance^14^. | Prostate cancer | Australia / New Zealand |
| 26 | Do not proceed with any treatment for localized prostate cancer unless the man has been offered a consultation with a urologist and a radiation oncologist and taken time to consider the advantages and disadvantages of each treatment option^14^. | Prostate cancer | Australia / New Zealand |
| 27 | Don’t treat with radioiodine low risk differentiated thyroid carcinomas (namely “microcarcinomas” or carcinomas <1 cm, in the absence of unfavorable prognostic factors), after total thyroidectomy^10^. | Thyroid cancer | Italy |
| **Treatment – Surgery (n=15)** | | | |
| 28 | Don’t routinely use extensive locoregional therapy in most cancer situations where there is metastatic disease and minimal symptoms attributable to the primary tumor (e.g., colorectal cancer)^11-13^. | Cancer | Canada |
| 29 | Do not routinely use extensive locoregional therapy in most cancer situations where there is metastatic disease and minimal symptoms attributable to the primary tumor^6^. | Cancer | Brazil |
| 30 | Don’t use surgery as the initial treatment without considering presurgical (neoadjuvant) systemic and/or radiation for cancer types and stage where it is effective at improving local cancer control, quality of life or survival^37^. | Cancer | USA / CC |
| 31 | Do not use surgery as the initial treatment without considering presurgical (neoadjuvant) systemic therapy and/or radiation for certain cancer types and stages where it is effective at improving local cancer control, quality of life, or survival^35^. | Cancer | Africa |
| 32 | Do not use surgery as the initial treatment without considering presurgical (neoadjuvant) systemic therapy and/or radiation for certain cancer types and stages where it is effective at improving local cancer control, quality of life or survival^5^. | Cancer | Philippinnes |
| 33 | Do not use surgery as the initial treatment without considering presurgical (neoadjuvant) systemic and/or radiation for cancer types and stage where it is effective at improving local cancer control, quality of life or survival^3,6^. | Cancer | Brazil, USA |
| 34 | Do not routinely re-operate on patients with invasive cancer if the cancer is close to the edge of the excised lumpectomy tissue^3,15,31^. | Breast cancer | USA / ASBrs |
| 35 | Don’t routinely use sentinel node biopsy in clinically node negative women ≥70 years of age with early-stage hormone receptor positive, HER2 negative invasive breast cancer^3,17,32^. | Breast cancer | USA / SSO |
| 36 | Do not routinely excise all the lymph nodes beneath the arm in patients having lumpectomy for breast cancer^15,31^. | Breast cancer | USA / ASBrS |
| 37 | Do not perform axillary dissection in clinical stages I and II of breast cancer if the lymph nodes are clinically normal, without first performing a sentinel lymph node biopsy^3,21^. | Breast cancer | Switzerland |
| 38 | Don’t perform axillary lymph node dissection for clinical stages I and II breast cancer with clinically negative lymph nodes without attempting sentinel node biopsy^28^. | Breast cancer | USA / ACS |
| 39 | Do not routinely perform a double mastectomy in patients who have a single breast with cancer^3,15,31^. | Breast cancer | USA / ASBrS |
| 40 | Contralateral prophylactic mastectomy (CPM) is not recommended for average risk women with early-stage unilateral breast cancer^11^. | Breast cancer | Canada |
| 41 | Don’t perform surgery to remove a breast lump for suspicious findings unless needle biopsy cannot be done^37^. | Breast cancer | USA / CC |
| 42 | Do not perform surgery to remove a breast lump without histologic confirmation of malignancy unless a needle biopsy cannot be performed^35^. | Breast cancer | Africa |
| **Treatment – Chemotherapy (n=11)** | | | |
| 43 | Do not forget to discuss the value of biomarker testing for specific solid tumours where targeted treatments have proven benefits^5^. | Cancer | Philippines |
| 44 | The original Choosing Wisely Statement #10 recommended against using a targeted therapy intended for use against a specific genetic aberration unless a patient’s tumor cells have a specific biomarker that predicts an effective response to the targeted therapy. While this statement continues to hold true, it is also currently being incorporated into a forthcoming ASCO Choosing Wisely 2021, which will update the currency and provide further context for this recommendation^27^. | Cancer | USA / ASCO |
| 45 | Do not use a targeted therapy intended for use against a specific genetic aberration unless a patient’s tumor cells have a specific biomarker that predicts an effective response to the targeted therapy^6^. | Cancer | Brazil |
| 46 | No molecular targeted therapy if a predictive biomarker has not been detected in the tumor^21^. | Cancer | Switzerland |
| 47 | Do not use bevacizumab for its various oncologic indications^9^. | Cancer | Italy |
| 48 | Don’t prescribe antibiotics to prevent infectious complications from neutropenia in cancer patients treated with standard dose chemotherapy^9^. | Cancer | Italy |
| 49 | Do not prescribe neither chemotherapy nor radiotherapy in the treatment of ductal carcinoma in situ of the breast^3,9^. | Breast cancer | Italy |
| 50 | Don’t use combination cytotoxic chemotherapy (multiple drugs) instead of chemotherapy with one drug when treating an individual for metastatic breast cancer unless the patient needs a rapid response to relieve tumor-related symptoms^3,27^. | Breast cancer | USA / ASCO |
| 51 | Do not use combination chemotherapy (multiple drugs) instead of chemotherapy with 1 (single) drug when treating an individual for metastatic breast cancer unless the patient needs a rapid response to relieve tumor-related symptoms^35^. | Breast cancer | Africa |
| 52 | Do not use combination cytotoxic chemotherapy when treating an individual for metastatic breast cancer unless the patient needs a rapid response to relieve tumour-related symptoms; instead, use a single cytotoxic agent^5^. | Breast cancer | Philippinnes |
| 53 | In the absence of EGFR mutations in non-small cell lung cancer, the use of tyrosine kinase inhibitors (Anti-EGFR) is not recommended^7^. | Lung cancer | Latin America |
| **Treatment – Radiation Therapy (n=40)** | | | |
| 54 | Do not routinely use extended fractionation schemes (N10 fractions) for palliation of bone metastases^38^. | Cancer | USA / ASTRO |
| 55 | Do not routinely use extended fractionation schemes (> 5 fractions) for palliation of uncomplicated bone metastases^6^. | Cancer | Brazil |
| 56 | Don’t routinely use more than one fraction for palliation of non-complex bone metastases^14^. | Cancer | Australia / New Zealand |
| 57 | Do not systematically use prolonged fractionation programs (more than ten sessions) in the palliative treatment of bone metastases^21^. | Cancer | Switzerland |
| 58 | Don’t recommend more than a single fraction of palliative radiation for an uncomplicated painful bone metastasis^39^. | Cancer | USA / AAHPM |
| 59 | Don’t recommend more than a single fraction of palliative radiation for an uncomplicated painful bone metastasis^11-13^. | Cancer | Canada |
| 60 | Choose not to prescribe more than a fraction of radiotherapy for palliation of uncomplicated painful bone metastases^16^. | Cancer | Portugal |
| 61 | Do not initiate longer courses of radiation therapy where evidence supports the use of shorter courses of radiation. For example, use a single fraction of palliative radiation for an uncomplicated painful bone metastasis and use shorter courses as a part of breast conservation therapy in women with early-stage invasive breast cancer^35^. | Cancer | Africa |
| 62 | Patients should avoid traveling abroad for carbon ion beam therapy without consulting a radiation oncologist or if it is not recommended by a multidisciplinary tumor board that includes a radiation oncologist^36^. | Cancer | Korea |
| 63 | Do not prescribe whole brain radiation over stereotactic radiosurgery (SRS) for patient with limited brain metastases (≤4 lesions) and good performance status^11,13^. | Cancer | Canada |
| 64 | Do not systematically add total brain radiotherapy to stereotaxic radiosurgery for limited brain metastases^21^. | Cancer | Switzerland |
| 65 | Don't routinely add adjuvant whole-brain radiation therapy to stereotactic radiosurgery for limited brain metastases^14^. | Cancer | Australia / New Zealand |
| 66 | Do not routinely apply adjunctive holocranial radiotherapy when the patient has undergone stereotactic radiotherapy^7^. | Cancer | Latin America |
| 67 | Choose not to routinely prescribe holocranial radiotherapy in addition to radiosurgery in patients with a limited number of brain metastases^16^. | Cancer | Portugal |
| 68 | Do not define a treatment program that includes radiation therapy without the involvement of the radiation oncologist from the beginning (usually, at time of the tumor first diagnosis) in the definition of the program^9^. | Cancer | Italy |
| 69 | Do not recommend the use of “special” techniques or radiation therapy machines without having previously obtained the recommendation by a radiation oncologist^9^. | Cancer | Italy |
| 70 | Do not use advanced radiation techniques where conventional radiation can be just as effective^34^. | Cancer | India |
| 71 | Choose not to initiate radiotherapy with non-curative intent without first defining treatment objectives with the patient^16^. | Cancer | Portugal |
| 72 | Do not neglect to offer radiotherapy delivered with hypofractionated schedules when the scientific evidence is already available^9^. | Cancer | Italy |
| 73 | Do not use to the extent possible, prolonged radiation therapy for palliative treatments in frail patients with a short life expectancy^9^. | Cancer | Italy |
| 74 | Do not prescribe protracted courses of radiotherapy when there is evidence to support equivalence of a hypofractionated or shorter fraction regimen^11^. | Cancer | Canada |
| 75 | Do not book multi-da patient visits for radiation treatments when these can be coordinated into a single trip^11^. | Cancer | Canada |
| 76 | Do not use single use disposable items when recyclabel or reusable alternatives exist in clinics and brachytherapy departments^11^. | Cancer | Canada |
| 77 | Do not initiate whole-breast radiation therapy as a part of breast conservation therapy in women age ≥50 with early-stage invasive breast cancer without considering shorter treatment schedules^38^. | Breast cancer | USA / ASTRO |
| 78 | Do not initiate whole-breast radiotherapy in 25 fractions as a part of breast conservation therapy in women aged 50 years with early-stage invasive breast cancer without considering shorter treatment schedules^34^. | Breast cancer | India |
| 79 | Don’t initiate whole-breast radiation therapy as a part of breast conservation therapy in women age ≥50 years with early-stage invasive breast cancer without considering shorter treatment schedulessa^3,14^. | Breast cancer | Australia / New Zealand |
| 80 | Do not start whole breast radiotherapy as part of breast-conserving treatment in women aged 50 years and older with early-stage invasive breast cancer, without considering shorter treatment programmes^21^. | Breast cancer | Switzerland |
| 81 | Don’t initiate whole breast radiotherapy in 25 fractions as a part of breast conservation therapy in women age ≥ 50 with early-stage invasive breast cancer without considering shorter treatment schedules^11,13^. | Breast cancer | Canada |
| 82 | Choose not to propose radiotherapy treatments, as an integral part of the conservative therapy of patients (aged 50 years or over) with invasive breast carcinoma in the early stages, without considering hypofractionation schemes^16^. | Breast cancer | Portugal |
| 83 | Do not initiate longer courses of radiation therapy where evidence supports the use of shorter courses of radiation. For example, use a single fraction of palliative radiation for an uncomplicated painful bone metastasis and use shorter courses as a part of breast conservation therapy in women with early-stage invasive breast cancer^35^. | Breast cancer | Africa |
| 84 | Do not routinely use intensity modulated radiation therapy to deliver whole-breast radiation therapy as part of breast conservation therapy^38^. | Breast cancer | USA / ASTRO |
| 85 | Choose not to routinely prescribe Intensity Modulated Radiotherapy (IMRT) in the management of breast cancer as an integral part of conservative treatment^38^. | Breast cancer | Portugal |
| 86 | Choose not to routinely replace brachytherapy with external radiotherapy in the therapeutic plan of patients with gynecological malignancies^16^. | Gynecological cancer | Portugal |
| 87 | Choose not to propose adjuvant radiotherapy for low-risk endometrial cancer (according to unknown molecular classification)^16^. | Endometrial cancer | Portugal |
| 88 | Do not recommend radiotherapy following hysterectomy for patients with low-risk endometrial cancer^21^. | Endometrial cancer | Switzerland |
| 89 | For uterine cancer, brachytherapy should be used as appropriate with or without external beam radiation therapy (EBRT)^36^. | Endometrial cancer | Korea |
| 90 | Do not routinely recommend proton beam therapy for prostate cancer outside of a prospective clinical trial or registry^38^. | Prostate cancer | USA / ASTRO |
| 91 | Choose not to propose proton radiotherapy in the treatment of prostate cancer outside of a research trial^16^. | Prostate cancer | Portugal |
| 92 | Do not treat patients with inoperable early-stage non-small cell lung cancer (or with multiple comorbidities) without discussing stereotactic body radiotherapy as a part of the shared decision-making process^6^. | Lung cancer | Brazil |
| 93 | Choose not to routinely propose adjuvant radiotherapy in patients with non-small cell lung carcinoma who have undergone surgical excision with disease-free margins and limited lymphatic metastasis (N0-N1)^16^. | Lung cancer | Portugal |
|  |  |  |  |
| **PALLIATIVE CARE (n=23)** | | | |
| 1 | Don’t use cancer-directed therapy for solid tumor patients with the following characteristics: low performance status (3 or 4), no benefit from prior evidence-based interventions, and no strong evidence supporting the clinical value of further anti-cancer treatment^3,28^. | Cancer | USA / ASCO |
| 2 | Avoid chemotherapy and instead focus on symptom relief and palliative care in patients with advanced cancer unlikely to benefit from chemotherapy (e.g., performance status 3 or 4)^11-13^. | Cancer | Canada |
| 3 | Avoid chemotherapy and instead focus on symptom relief and palliative care in patients with advanced cancer that are unlikely to benefit from chemotherapy (Including one or more of the above: performance status 3 or 4, no benefit from previous evidence-based interventions, not suitable for a clinical trial, and no evidence supporting the clinical value of further anticancer treatment)^34^. | Cancer | India |
| 4 | Do not use cancer-directed therapy for patients with solid tumours with ALL of the following characteristics: low performance status (PS) (3 or 4), no benefit from prior evidence-based interventions and no strong evidence supporting the clinical value of further anti-cancer treatment. Instead, focus on symptom relief and palliative care^5^. | Cancer | Philippinnes |
| 5 | Do not use cancer-directed therapy for patients with solid tumors with the following characteristics: low performance status (3 or 4), no benefit from prior evidence-based interventions, not eligible for a clinical trial and no strong evidence supporting the clinical value of further anti-cancer treatment; place a focus on symptom relief and palliative care^6^. | Cancer | Brazil |
| 6 | Don’t routinely use cancer-directed therapy for solid tumor patients with low performance status (3 or 4) or progressive after 2-3 therapeutic lines but prioritize palliative care^9^. | Cancer | Italy |
| 7 | Do not use systemic therapy for solid-tumor patients with the following characteristics: low performance status (3 or 4), no benefit from prior evidence-based interventions, and no strong evidence supporting the clinical value of additional anticancer treatment; instead, focus on symptom relief and palliative care^35^. | Cancer | Africa |
| 8 | Do not use cancer-directed therapy in patients with solid tumours with low-performance status (3 or 4), no benefit from previous interventions, and not eligible for inclusion in clinical trials. Prioritise palliative treatment to relieve symptoms^7^. | Cancer | Latin America |
| 9 | Avoid cytotoxic chemotherapy in patients with advanced cancer who are unlikely to benefit from chemotherapy (ECOG performance status 3 or 4) and continue to focus on symptom relief and palliative care^14^. | Cancer | Australia |
| 10 | No tumor-targeted therapy in patients with performance status 3-4, after failure of standard therapies and lack of evidence for other lines of treatment^21^. | Cancer | Switzerland |
| 11 | In advanced cancer, the use of chemotherapy that is unlikely to be beneficial and may cause harm should be minimised^24^. | Cancer | United Kingdom |
| 12 | Do not propose any type of palliative chemotherapy in the end-of-life setting^9^. | Cancer | Italy |
| 13 | Don’t delay palliative care for a patient with serious illness who has physical, psychological, social or spiritual distress because they are pursuing disease-directed treatment^39^. | Cancer | USA / AAHPM |
| 14 | Don’t delay or avoid palliative care for a patient with metastatic cancer because they are pursuing disease-directed treatment^11-13^. | Cancer | Canada |
| 15 | Do not delay discussion of and referral to palliative care for a patient with serious illness just because they are pursuing disease-directed treatment^14^. | Cancer | Australia / New Zealand |
| 16 | Do not delay or avoid palliative care for a patient with metastatic cancer because they are pursuing disease-directed treatment^34^. | Cancer | India |
| 17 | Do not delay palliative care for a patient with serious illness who has physical, psychological, social, or spiritual distress because the patient is pursuing disease-directed treatment^34^. | Cancer | Africa |
| 18 | No delay in introducing palliative care for patients with a life-threatening illness (also non-oncological) who have physical, psychological, social or spiritual needs, simply because they are undergoing disease-modifying or life-prolonging treatment^21^. | Cancer | Switzerland |
| 19 | Don’t initiate or prolong artificial nutrition (enteral or parenteral) in late-stage cancer patients with a life expectancy of less than a few weeks and a Performance Status <50^9^. | Cancer | Italy |
| 20 | In patients with advanced disease or cancer who are suffering from malnutrition, do not systematically introduce artificial nutrition^21^. | Cancer | Switzerland |
| 21 | For patients with limited life expectancy (such as advanced cardiac, renal or respiratory failure, metastatic malignancy, third line chemotherapy) ensure patients have a ‘goals of care’ discussion at or prior to admission to ICU and for patients in ICU who are at high risk for death or severely impaired functional recovery, ensure that alternative care focused predominantly on comfort and dignity is offered to patients and their families^14^. | Cancer | Australia / New Zealand |
| 22 | Do not start anticancer treatment in patients with advanced/metastatic disease without defining the functional goals/benefits of treatment with the patient and without considering palliative care support^21^. | Cancer | Switzerland |
| 23 | Don’t delay the provision of palliative care for women with advanced or relapsed gynecologic cancer, including referral for specialty level palliative medicine^22^. | Gynecological cancer | USA / SGO |
|  |  |  |  |
| **SURVEILLANCE (n=29)** | | | |
| 1 | Do not order tests to detect recurrent cancer in asymptomatic patients if there is not a realistic expectation that early detection of recurrence can improve survival or quality of life^3, 11-13^. | Cancer | Canada |
| 2 | Do not order tests to detect recurrent cancer in asymptomatic patients if there is not a realistic expectation that early detection of recurrence can improve survival or quality of life (Including biomarkers, PET/CT and CT scans, and endoscopy and radionuclide scans)^34^. | Cancer | India |
| 3 | Do not order test to detect recurrent cancer in asymptomatic patients if there is not a realistic expectation that early detection of recurrence can improve survival or quality of life^35^. | Cancer | Africa |
| 4 | Avoid using PET or PET-CT scanning as part of routine follow-up care to monitor for a cancer recurrence in asymptomatic patients who have finished initial treatment to eliminate the cancer unless there is high-level evidence that such imaging will change the outcome^27^. | Cancer | USA / ASCO |
| 5 | Avoid biomarker testing and imaging for recurrent cancer in previously treated asymptomatic patients unless there is evidence that early detection of recurrence may improve survival or quality of life; including avoiding biomarkers or imaging in asymptomatic patients treated for breast cancer with curative intent^7^. | Cancer | Latin America |
| 6 | Avoid tests (biomarkers and imaging) for recurrent cancer in previously treated asymptomatic patients unless there is evidence that early detection of recurrence can improve survival or quality of life; including avoiding surveillance testing (biomarkers) or imaging (PET, CT and radionuclide bone scans) for asymptomatic individuals who have been treated for breast cancer with curative intent^14^. | Cancer | Australia |
| 7 | Don’t use whole-body scans for early tumor detection in asymptomatic patients^19^. | Cancer | USA / ACPM |
| 8 | Do not use whole body Positron Emission Tomography - Computed Tomography (PET-CT) scans to detect recurrence after completing curative treatment for asymptomatic patients with early-stage solid tumours^5^. | Cancer | Philippinnes |
| 9 | No PET in the follow-up of asymptomatic patients when there is complete remission after treatment, unless there is high evidence of the usefulness of a PET^21^. | Cancer | Switzerland |
| 10 | After treatment for cancer, the use of routine scanning should only be used where this is beneficial to the patient^24^. | Cancer | United Kingdom |
| 11 | Restrict the use of serum tumour marker tests to the monitoring of a cancer known to produce these markers. There may be a role, however, for tumour marker measurement in the initial investigation and assessment of high risk or symptomatic individuals^14^. | Cancer | Australia |
| 12 | Do not perform serum tumour marker tests except to evaluate or monitor a cancer known to produce these markers^14^. | Cancer | Australia |
| 13 | Don’t initiate surveillance testing after cancer treatment without providing the patient a survivorship care plan^37^. | Cancer | USA(CC) |
| 14 | Do not deliver care in a high-cost setting when it could be delivered just as effectively in a lower-cost setting^34^. | Cancer | India |
| 15 | Don’t deliver care (e.g., follow-up) in a high-cost setting (e.g., inpatient, cancer center) that could be delivered just as effectively in a lower-cost setting (e.g., primary care)^11-13^. | Cancer | Canada |
| 16 | The use of PET/CT is not recommended for the follow-up of patients undergoing palliative cancer treatment. It should not be routinely used to detect possible disease recurrence in patients who have completed cancer treatment, unless clinically suspected and/or suspected by imaging^7^. | Cancer | Latin America |
| 17 | Do not order PET/CT scans to monitor response to palliative chemotherapy^34^. | Cancer | India |
| 18 | Do not conduct in-person visits for oncology care if a virtual visit is feasible, safe, clinically appropriate (i.e. no physical exam required, not for delivery of bad news or major updates) and is preferred by the patient^11^. | Cancer | Canada |
| 19 | Don’t perform surveillance testing (biomarkers) or imaging (PET, CT, and radionuclide bone scans) for asymptomatic individuals who have been treated for breast cancer with curative intent^27^. | Breast cancer | USA / ASCO |
| 20 | Don’t perform laboratory tests (including biochemical profile), imaging (chest x-rays, liver and pelvic ultrasound, PET, CT and radionuclide bone scans) or serum cancer markers for asymptomatic patients after surgery for breast cancer, in the absence of clinical signs^9^. | Breast cancer | Italy |
| 21 | Do not perform colposcopy in patients treated for cervical cancer with Pap tests of low-grade squamous intraepithelial lesion (LGSIL) or less^22^. | Cervical cancer | USA / SGO |
| 22 | Don’t perform colposcopy in patients treated for cervical cancer with radiation unless high-grade changes are present^22^. | Cervical cancer | USA / SGO |
| 23 | Avoid routine imaging for cancer surveillance in women with gynecologic cancer, specifically ovarian, endometrial, cervical, vulvar, and vaginal cancer^22^. | Gynecological cancer | USA / SGO |
| 24 | Imaging for cancer surveillance in women with gynecologic cancer, specifically ovarian, endometrial, cervical, vulvar and vaginal cancer should be driven by symptoms/signs^22^. | Gynecological cancer | USA / SGO |
| 25 | Do not perform Pap tests for surveillance of women with a history of endometrial cancer^22^. | Cervical cancer | USA / SGO |
| 26 | Do not perform routine colonoscopic surveillance yearly in patients after colon cancer surgery; frequency should be based on previous colonoscopy findings and guidelines^7^. | Colorectal cancer | Latin America |
| 27 | Don’t perform routine colonoscopic surveillance every year in patients following their colon cancer surgery; instead, frequency should be based on the findings of the prior colonoscopy and corresponding guidelines^11-13^. | Colorectal cancer | Canada |
| 28 | Don’t obtain routine blood work (e.g., CBC, liver function tests) other than a CEA level for surveillance for colorectal cancer^17^. | Colorectal cancer | USA / SSO |
| 29 | Don’t perform routine PET-CT in the initial staging of localized colon or rectal cancer or as part of routine surveillance for patients who have been curatively treated for colon or rectal cancer^17^. | Colorectal cancer | USA / SSO |

*AGS* American Geriatrics Society*, AMDA* Society for Post-Acute and Long-Term Care Medicine*, SGIM* Society of General Internal Medicine*, PALTmed* Post-Acute and Long-Term Care Medical Association, *ASBrS* The American society of Breast Surgeons*, SSO* Society of Surgical Oncology*, AAFP* American Academy of Family Physicians, *ACPM* American College of Preventive Medicine, *ASCCP* American Society for Colposcopy and Cervical Pathology*, ACOG* The American College of Obstetricians and Gynecologists, *SGO* Society of Gynecologic Oncology*, ASCO* American Society of Clinical Oncology*, AGA* American Gastroenterological Association*, ACS* American College of Surgeons, *ATS* American Thoracic Society, *CC* Comission on Cancer,  *ASTRO American* Society for Radiation Oncology, *AAHPM* American Academy of Hospice and Palliative Medicine

| Cancer / Metastatic cancer | Breast cancer | Gynecological cancer | Prostate cancer | Colorectal cancer | Lung cancer, Melanoma  and Thyroid cancer |
| --- | --- | --- | --- | --- | --- |

**Bibliography**

1. Murphy J, Tanner T, Komorowski J. Shared Decision-Making withChoosing Wisely. Vol. 23, Nursing for Women’s Health. Elsevier B.V.; 2019. p. 253–64.

2. Post-Acute and Long-Term Care Medical Association. Fifteen Things Physicians and Patients Should Question. [Internet]. 2015 [cited 2024 Jul 29]. Available from: https://paltc.org/programs/choosing-wisely

3. Shah H, Surujballi J, Awan AA, Hutton B, Arnaout A, Shorr R, et al. A scoping review characterizing “Choosing Wisely®” recommendations for breast cancer management. Breast Cancer Res Treat. 2021 Feb 1;185(3):533–47.

4. American Geriatrics Society.  Ten Things Physicians and Patients Should Question. [Internet]. 2014 [cited 2024 Jul 29]. Available from: https://www.healthinaging.org/choosing-wisely

5. Ting FI, Uy CD, Bebero KG, Sacdalan DB, Abarquez HS, Nilo G, et al. Choosing Wisely Philippines: ten low-value or harmful practices that should be avoided in cancer care. Ecancermedicalscience. 2022;16:1–8.

6. de Moraes FY, Marta GN, Mitera G, Forte DN, Pinheiro RN, Vieira NF, et al. Choosing Wisely for oncology in Brazil: 10 recommendations to deliver evidence-based cancer care. Nat Med. 2022 Sep 1;28(9):1738–9.

7. Ismael J, Esandi E, Arroyo G, Becerra S, Bejarano S, Castro C, et al. Choosing Wisely in oncology in Latin America: what SLACOM does not recommend in the care of cancer patients in Latin America. Ecancermedicalscience. 2024;18.

8. Tokuda Y. Current Status of Choosing Wisely in Japan. General Medicine. 2015;16(1):3–4.

9. Choosing Wisely Italy, Raccomandazioni [Internet]. 2021 [cited 2024 Jul 18]. Available from: https://choosingwiselyitaly.org/en/raccomandazioni/

10. Italian Association of Nuclear Medicine and Molecular Imaging. Choosing Wisely Italy. 2015 [cited 2024 Jul 31]; Available from: https://choosingwiselyitaly.org/en/societa/aimn-2/

11. Choosing Wisely Canada. Recommendations and resources for clinicians by healthy specialty [Internet]. 2024 [cited 2024 Jul 18]. Available from: https://choosingwiselycanada.org/recommendations/

12. Mitera G, Earle C, Latosinsky S, Booth C, Bezjak A, Desbiens C, et al. Choosing Wisely Canada Cancer List: Ten Low-Value or Harmful Practices That Should Be Avoided In Cancer Care. J Oncol Pract. 2015;11(3):e296–303.

13. Karim S, Doll CM, Dingley B, Merchant SJ, de Moraes FY, Booth CM. The Choosing Wisely Oncology Canada Cancer List: An Update. J Cancer Policy. 2023 Sep 1;37.

14. Choosing Wisely Australia. Recommendations. [Internet]. 2022 [cited 2024 Jul 29]. Available from: https://www.choosingwisely.org.au/recommendations

15. The American Society of Breast Surgeons.  Five Things Physicians and Patients Should Question [Internet]. 2018 [cited 2024 Jul 29]. Available from: https://www.breastsurgeons.org/resources/choosing_wisely

16. Choosing Wisely Portugal. Escolhas criteriosas em saúde. [Internet]. [cited 2024 Jul 19]. Available from: https://ordemdosmedicos.pt/choosing-wisely-portugal-escolhas-criteriosas-em-saude

17. Society of Surgical Oncology. Five Things Physicians and Patients Should Question. [Internet]. 2020 [cited 2024 Jul 29]. Available from: https://www.surgonc.org/wp-content/uploads/2020/11/SSO-5things-List_2020-Updates-11-2020.pdf

18. American Academy of Family Physicians. Encouraging conversations between physicians and patients to improve care. [Internet]. [cited 2024 Jul 29]. Available from: https://www.aafp.org/family-physician/patient-care/clinical-recommendations/choosing-wisely.html

19. Livingston CJ, Freeman RJ, Mohammad A, Costales VC, Titus TM, Harvey BJ, et al. Choosing Wisely® in preventive medicine the American College of Preventive Medicine’s top 5 list of recommendations. Am J Prev Med. 2016 Jul 1;51(1):141–9.

20. The American College of Obstetricians and Gynecologists. Five More Things Physicians and Patients Should Question" in obstetrics-gynecology [Internet]. 2016 [cited 2024 Jul 29]. Available from: https://www.acog.org/practice-management/patient-safety-and-quality/partnerships/choosing-wisely

21. Smarter Medicine. Choosing Wisely Switzerland. [Internet]. 2024 [cited 2024 Jul 19]. Available from: https://www.smartermedicine.ch/de/top-5-listen/radio-onkol ogie.html

22. Society of Gynecologic Oncology.  Five Things Physicians and Patients Should Question. [Internet]. 2022 [cited 2024 Jul 29]. Available from: https://www.sgo.org/wp-content/uploads/2023/12/Choosing-Wisely-Five-Tips-for-a-Meaningful-Conversation-Between-Patients-and-Providers.pdf

23. Gemeinsam gut entscheidn. [Internet]. 2019 [cited 2024 Jul 19]. Available from: https://www.gemeinsam-gut-entscheidn.at/

24. Choosing Wisely UK. Recommendations for clinicians 2016/18 2019 [Internet]. [cited 2024 Jul 19]. Available from: https://choosingwisely.co.uk/recommendations-archive/

25. Glechner A, Rabady S, Bachler H, Dachs C, Flamm M, Glehr R, et al. A Choosing Wisely top-5 list to support general practitioners in Austria. Wiener Medizinische Wochenschrift. 2021 Oct 1;171(13–14):293–300.

26. בוחרים בתבונה [Internet]. 2017 [cited 2024 Jul 31]. Available from: https://www.ima.org.il/MedicineQuality/ChoosingWisely.aspx

27. American Society of Clinical Oncology Choosing Wisely. [Internet]. 2021 [cited 2024 Jul 29]. Available from: https://society.asco.org/news-initiatives/current-initiatives/cancer-care-initiatives/value-cancer-care/choosing-wisely

28. American College of Surgeons. Five Things Physicians and Patients Should Question. [Internet]. [cited 2024 Jul 29]. Available from: https://www.facs.org/media/xp3msgu0/acslist.pdf

29. Wiener RS, Ouellette DR, Diamond E, Fan VS, Maurer JR, Mularski RA, et al. An Official American Thoracic Society/American College of Chest Physicians Policy statement: The choosing wisely top five list in adult pulmonary medicine. Chest. 2014;145(6):1383–91.

30. Gupta S, Goodridge D, Pakhalé S, McIntyre K, Pendharkar SR. Choosing wisely: The Canadian Thoracic Society’s list of six things that physicians and patients should question. Canadian Journal of Respiratory, Critical Care, and Sleep Medicine. 2017 Apr 3;1(2):54–61.

31. Landercasper J, Bailey L, Berry TS, Buras RR, Degnim AC, Fayanju OM, et al. Measures of Appropriateness and Value for Breast Surgeons and Their Patients: The American Society of Breast Surgeons Choosing Wisely ®Initiative. Ann Surg Oncol. 2016 Oct 1;23(10):3112–8.

32. Trooboff SW, Kang R, Margenthaler J, Wong SL. Choosing Wisely: Optimizing Routine Workup for the Newly Diagnosed Breast Cancer Patient. Curr Breast Cancer Rep. 2018 Jun 1;10(2):62–73.

33. Eskander A, Monteiro E, O’Connell D, Taylor SM. Head and Neck Surgical Oncology Choosing Wisely Campaign: Imaging for patients with hoarseness, fine needle aspiration for neck mass, and ultrasound for odynophagia. Journal of Otolaryngology - Head and Neck Surgery. 2018 Jan 8;47(1).

34. Pramesh CS, Chaturvedi H, Reddy VA, Saikia T, Ghoshal S, Pandit M, et al. Choosing Wisely India: ten low-value or harmful practices that should be avoided in cancer care. Lancet Oncol. 2019 Apr 1;20(4):e218–23.

35. Rubagumya F, Mitera G, Ka S, Manirakiza ; Achille, Decuir P, Msadabwe SC, et al. Choosing Wisely Africa: Ten Low-Value or Harmful Practices That Should Be Avoided in Cancer Care. JCO Global Oncol [Internet]. 2020;6:1192–9. Available from: https://ascopubs.org/go/authors/open-access

36. Kim JY. Choosing Wisely, The Korean Perspective: The Launch of the Nationwide “Right Decision in Cancer Care” Initiative. Int J Radiat Oncol Biol Phys. 2020 Jul 1;107(3):602–3.

37. Comission on Cancer.  Five Things Physicians and Patients Should Question [Internet]. [cited 2024 Jul 29]. Available from: https://www.facs.org/media/wegncq3f/coclist.pdf

38. Hahn C, Kavanagh B, Bhatnagar A, Jacobson G, Lutz S, Patton C, et al. Choosing Wisely: The American Society for Radiation Oncology’s Top 5 list. Pract Radiat Oncol. 2014 Nov 1;4(6):349–55.

39. American Academy of Hospice and Palliative Medicine.  Five Things Physicians and Patients Should Question in Hospice and Palliative Medicine. [Internet]. [cited 2024 Jul 29]. Available from: https://aahpm.org/outreach/choosing-wisely
